# Tribbles homolog 3 denotes a poor prognosis in breast cancer and is involved in hypoxia response

**DOI:** 10.1186/bcr2934

**Published:** 2011-08-24

**Authors:** Marloes Wennemers, Johan Bussink, Blanca Scheijen, Iris D Nagtegaal, Hanneke WM van Laarhoven, James A Raleigh, Mahesh A Varia, Joop JTM Heuvel, Kasper M Rouschop, Fred CGJ Sweep, Paul N Span

**Affiliations:** 1 Department of Laboratory Medicine, Radboud University Nijmegen Medical Centre, PO Box 9101, 6500 HB Nijmegen, The Netherlands; 2 Department of Radiation Oncology, Radboud University Nijmegen Medical Centre, PO Box 9101, 6500 HB Nijmegen, The Netherlands; 3 Department of Pediatrics, Laboratory of Pediatric Oncology, Radboud University Nijmegen Medical Centre, PO Box 9101, 6500 HB Nijmegen, The Netherlands; 4 Department of Pathology, Radboud University Nijmegen Medical Centre, PO Box 9101, 6500 HB Nijmegen, The Netherlands; 5 Department of Medical Oncology, Radboud University Nijmegen Medical Centre, PO Box 9101, 6500 HB Nijmegen, The Netherlands; 6 Department of Radiation Oncology, UNC School of Medicine, 333 South Columbia Street NC27514 Chapel Hill, NC, USA; 7 Maastricht Radiation Oncology (MaastRo) Lab, GROW-School for Oncology and Developmental Biology, University of Maastricht, PO box 616, 6200 AP Maastricht, The Netherlands

## Abstract

**Introduction:**

Hypoxia in solid tumors is associated with treatment resistance, resulting in poor prognosis. Tribbles homolog 3 (TRIB3) is induced during hypoxia and is involved in multiple cellular pathways involved in cell survival. Here, we investigated the role of *TRIB3 *in breast cancer.

**Methods:**

*TRIB3 *mRNA expression was measured in breast tumor tissue from 247 patients and correlated with clinicopathological parameters and clinical outcome. Furthermore, we studied TRIB3 expression regulation in cell lines, xenografts tissues and human breast cancer material using Reverse transcriptase, quantitative polymerase chain reaction (RT-qPCR) and immunohistochemical staining. Finally, the effect of small interfering RNA (siRNA) mediated *TRIB3 *knockdown on hypoxia tolerance was assessed.

**Results:**

Breast cancer patients with low, intermediate or high *TRIB3 *expression exhibited a mean disease free survival (DFS) of 80 (95% confidence interval [CI] = 74 to 86), 74 (CI = 67 to 81), and 63 (CI = 55 to 71) months respectively (*P *= .002, Mantel-Cox log-rank). The prognostic value of *TRIB3 *was limited to those patients that had received radiotherapy as part of their primary treatment (*n *= 179, *P *= .005) and remained statistically significant after correction for other clinicopathological parameters (DFS, Hazard Ratio = 1.90, CI = 1.17 to 3.08, *P *= .009). In breast cell lines *TRIB3 *expression was induced by hypoxia, nutrient starvation, and endoplasmic reticulum stress in an hypoxia inducible factor 1 (HIF-1) independent manner. *TRIB3 *induction after hypoxia did not increase with decreasing oxygen levels. In breast tumor xenografts and human breast cancer tissues TRIB3 co-localized with the hypoxic cell marker pimonidazole. The induction of *TRIB3 *by hypoxia was shown to be regulated via the PERK/ATF4/CHOP pathway of the unfolded protein response and knockdown of *TRIB3 *resulted in a dose-dependent increase in hypoxia sensitivity.

**Conclusions:**

*TRIB3 *is independently associated with poor prognosis of breast cancer patients, possibly through its association with tumor cell hypoxia.

## Introduction

The accelerated growth and erratic angiogenesis of solid tumors induce a lack of oxygen and nutrients in parts of the tumor that are distal to functional blood vessels. This is known to have important repercussions for treatment sensitivity and prognosis of cancer patients [1-3]. Variation in the duration and severity of hypoxic stress differentially activates different "do or die" programs and leads to substantial phenotypic variations amongst otherwise identical tumor cells. Best known in this perspective is the Hypoxia-Inducible Factor 1 (HIF-1) pathway, which is induced during hypoxia and has often been associated with poor prognosis in solid tumors including breast cancer [4-6]. Under more anoxic conditions another hypoxia-related program is activated, that is the unfolded protein response (UPR) [7], which results from endoplasmic reticulum (ER) stress caused by misfolding of proteins in the ER. One of the mechanisms that are activated by the UPR is autophagy [8], which can temporarily relieve ER stress by reutilization of cellular components. ER stress-induced expression of activating transcription factor 4 (ATF4) and CHOP (C/EBP homologous protein) also leads to transcription of Tribbles homolog 3 (TRIB3) [9]. TRIB3 is known to inhibit phosphorylation of AKT/protein kinase B (AKT) [10]. AKT is a phosphoinositide-dependent serine/threonine protein kinase that plays a critical role in the signal transduction of growth factor receptors. Furthermore, TRIB3 has been described to have a role in the mitogen activated protein kinase (MAPK) pathway [11] and nuclear factor (NF) κB activated apoptosis [7]. However, the literature is not conclusive on the role of TRIB3 in cell fate; it has been described to have pro-apoptotic [9] as well as anti-apoptotic features [12]. TRIB3 is strongly upregulated by hypoxia, nutrient starvation and ER stress-inducing agents [13-15] and has been implicated in ER stress-induced autophagy [16]. Interestingly, TRIB3 was shown to be upregulated compared with normal tissue in tumors of the human lung, colon, and breast [14,17,18].

We hypothesized that TRIB3 could be relevant for breast cancer prognosis, because in breast cancer, several ER stress, UPR, and/or hypoxia-associated markers have been found to be related to prognosis [19-22]. Furthermore, the involvement of TRIB3 in the other pathways described above makes it more interesting than other proteins in the UPR and other hypoxia pathways, because, for example, the growth factor receptor-induced phosphorylation cascades are also known to be relevant for breast cancer treatment [23]. We hypothesized that TRIB3 could be an important protein by determining the tumor cell fate under stressed conditions. To this end, *TRIB3 *mRNA expression levels were correlated with disease characteristics and patient survival in a large breast cancer patient cohort. In addition, we determined if *TRIB3 *expression could be induced in breast cancer cells by a variety of stressors, and we assessed the expression and localization of TRIB3 in breast cancer xenografts and patient tissues. Finally, we determined the effect of *TRIB3 *knockdown on the hypoxia response of breast cancer cells.

## Materials and methods

### Patient samples

Coded tumor tissues were used in accordance with the Code of Conduct of the Federation of Medical Scientific Societies in the Netherlands ('Code for Proper Secondary Use of Human Tissue in the Netherlands' [24]). The study adhered to all relevant institutional and national guidelines, and was reported according to REMARK guidelines [25]. A series of 247 patients with unilateral, resectable breast cancer who had undergone resection of their primary tumor between November 1987 and December 1997 were selected based on the availability of frozen tissue in the tumor bank of the Department of Laboratory Medicine of the Radboud University Nijmegen Medical Centre. This bank contains frozen tumor tissue from breast cancer patients of seven different hospitals of the Comprehensive Cancer Centre East in the Netherlands. The patients, inclusion and exclusion criteria, and their treatment have been described earlier [26]. To summarize, the median age was 59 years (range 31 to 88 years). Patients had undergone modified radical mastectomy (*n *= 178) or a breast-conserving lumpectomy (*n *= 69). Postoperative radiotherapy to the breast after an incomplete resection or after breast-conserving treatment, or parasternal radiotherapy when the tumor was medially localized, had been administered to 179 patients. Adjuvant systemic therapies were given almost exclusively to the 128 patients with lymph node involvement, according to standard practice at that time [26]. During follow up, 95 patients experienced a recurrence, either local or distant. A representative part of each tumor was macroscopically selected by a pathologist. The material was frozen in liquid nitrogen and determination of estrogen receptor and progesterone receptor status was performed by ligand binding assay according to the dextran-charcoal method [27]. Aliquots of tissue were pulverized using a microdismembrator (Braun, Melsungen, Germany) and kept in liquid nitrogen until RNA isolation.

To obtain patient material containing the exogenous hypoxia marker pimonidazole, patients with newly diagnosed breast cancer were enrolled in the period 1997 to 1999 in a tumor hypoxia study in accordance with a research protocol approved by the Institutional Review Board at the University of North Carolina Hospitals. The patients provided signed informed consent prior to their participation in the study. Prior to tumor biopsy, patients received an intravenous infusion of pimonidazole hydrochloride (0.5 g/m^2^, Hypoxyprobe-1™, NPI Inc, Belmont, MA, USA) diluted in 100 ml NaCl 0.9% for 20 minutes. Between 16 to 24 hours later, biopsies were obtained from primary tumors. After biopsy, fresh tumor samples were placed in cold 10% neutral buffered formalin, held at 4°C for 12 to 24 hours, and processed into paraffin blocks. Four *μ*m thick sections were sectioned and mounted on glass slides in preparation for immunohistochemical staining. One slide per block was stained with hematoxylin and eosin for pathologic review to confirm the presence of tumor. Based on the hypoxia scoring of the tumors according to a calibrated scoring system [28] three tumors with a high percentage of hypoxia (> 15% of the viable area) were chosen for analysis of TRIB3 expression.

### Xenograft tissue

Xenografts of MDA-MB-231 cells were obtained after subcutaneous injection of 1 × 10^6 ^cells suspended in RPMI (MP Biomedicals, Illkirch, France) in six-week-old female athymic mice (BALB/c nu/nu, BonholdGard, Denmark). Animal housing and experimental procedures were in accordance with international guidelines and approved by the local ethical committee for animal use, respectively. At a mean tumor diameter of 6 to 8 mm at approximately six weeks after seeding that time mice were injected intravenously with 0.1 ml of 0.9% NaCl containing 2 mg of the hypoxic cell marker pimonidazole hydrochloride (1-((2-hydroxy-3-piperidinyl)propyl)-2-nitroimidazole hydrochloride, Natural Pharmaceuticals, Inc., Research Triangle Park, NC, USA) 60 minutes before harvesting the tumors. Pimonidazole is a bioreductive chemical probe with an immuno-recognizable side chain, which was described previously as a marker for hypoxia [29-31]. The animals were killed by cervical dislocation and the harvested xenograft tissues were immediately frozen in liquid nitrogen.

### Cell lines

MCF7 and MDA-MB-231 (ATCC, LGC Promochem, London, UK) human breast cancer cells were cultured for a limited number of passages in standard culture medium ((DMEM, MP Biomedicals, Amsterdam, the Netherlands) with 10% dialyzed FCS (Invitrogen, Breda, the Netherlands), 2 mM L-glutamine, 20 mM HEPES, 10 U/ml penicillin, 10 μg/ml streptomycin, and nonessential amino acids (NEAA, MP Biomedicals)) at 37°C with 5% CO_2_, unless stated otherwise. Knockdown of *TRIB3 *was performed using siRNA transfection reagent SAINT-RED (Synvolux Therapeutics BV, Groningen, the Netherlands). siRNAs MISSION^® ^siRNA Universal Negative Control #1 (SIC001), TRIB3 (1) (SASI_Hs01_00197510) and TRIB3 (2) (SASI_Hs01_00197511) were acquired from Sigma-Aldrich (St. Louis, MO, USA). The knockdown of HIF-1α, PRKR-like endoplasmic reticulum kinase (PERK), inositol-requiring 1 (IRE1), activating transcription factor 6 (ATF6), ATF4 and CHOP was performed in MCF7 cells as described previously for HCT116 cells [32].

### Detection of RNA

Total RNA was isolated with the RNeasy RNA isolation kit (Qiagen, Hilden, Germany) with on-column DNAse treatment. For the reverse transcriptase quantitative PCR (RT-qPCR) Reverse Transcription System from Promega Benelux B.V. (Leiden, the Netherlands) was used and cDNAs were amplified with specific primers (*TRIB3 *forward: att agg cag ggt ctg tcc tgt g, reverse: agt atg gac ctg gga ttg tgg a; *VEGF *forward: ccg cag acg tgt aaa tgt tcc t, reverse:cgg ctt gtc aca tct gca agt a; *PAI-1 *forward:ggc cat gga aca agg atg aga, reverse:gac cag ctt cag atc ccg ct; *CAIX *forward:gag gcc tgg ccg tgt tg, reverse:aat cgc tga gga agg ctc ag; *MIF *forward:cag ccc gga cag ggt cta c, reverse:tct tag gcg aag gtg gag ttg; *ATF4 *forward: cct tca cct tct tac aac c, reverse: gta gtc tgg ctt cct atc t; *GRP78/BiP *forward:tct atg aag gtg aaa gac cc, reverse:ctg tca ctc gaa gaa tac ca) using Sybr Green Master Mix (Applied Biosystems, Nieuwerkerk a/d lJssel, the Netherlands) on an ABI Prism 7700 Sequence detection system (Applied Biosystems, Nieuwerkerk a/d lJssel, the Netherlands). All samples were normalized for levels of hypoxanthine-guanine phosphoribosyltransferase (HPRT) expression. In the experiment in which the pathways involved in TRIB3 regulation were determined, RNA was reverse-transcribed using I-Script (Bio-Rad Laboratories BV, Veenendaal, the Netherlands). Furthermore, these samples were normalized to 18S rRNA.

### Detection of protein

Protein localization of TRIB3 and other relevant proteins or markers in xenograft and human breast cancer tissue was determined using immunohistochemical staining. The frozen xenografts or frozen tumor tissues were sectioned at 5 μm thickness after embedding in Tissue-Tek (Sakura Finetek Europe, Zoeterwoude, the Netherlands). Sections were mounted on poly-L-Lysine coated microscopic slides (Menzel, Braunschweig, Germany), fixated in acetone and rehydrated in 0.1 M phosphate buffered saline pH 7.4 (PBS, Klinipath, Duiven, the Netherlands). Between all antibody incubations, sections were rinsed three times in PBS (Klinipath, Duiven, the Netherlands). The following primary antibodies were dissolved in primary antibody diluent (PAD, AbD serotec, Oxford, UK) and stained with the appropriate labeled secondary antibodies; rabbit-anti-TRIB3 (Calbiochem, San Diego, CA, USA), PAL-E for blood vessels in human tissue (Euro Diagnostica, Arnhem, the Netherlands), rat monoclonal against mouse endothelium (9F1, kind gift from Dr. G. van Muijen of the Department of Pathology, Radboud University Nijmegen Medical Centre, Nijmegen, the Netherlands), and rabbit-anti-pimonidazole for hypoxic regions in xenograft tissue. Slides were enclosed using Fluorostab (ICN pharmaceuticals, Inc. Zoetermeer, the Netherlands). Paraffin embedded breast tissue sections (4 μm) were deparaffinized and re-hydrated in histosafe (Klinipath, Duiven, the Netherlands) and graded alcohols (100%-96%-70%). For antigen retrieval, slides were heated (90°C) in 10 mM citrate buffer pH 6.0 (DAKO, Glostrup, Denmark). Endogenous peroxidase was blocked with 3% H_2_O_2 _in methanol before the incubation with polyclonal rabbit-anti-TRIB3 (Novus Biologicals, Cambridge, UK) or rabbit-anti-pimonidazole. Subsequently, slides were incubated with Powervision (Lab Vision Products, Thermo Fisher Scientific, Cheshire, UK) and visualization of peroxidase was performed using PowerDAB (Lab Vision Products, Thermo Fisher Scientific, Cheshire, UK). A counterstain with hematoxylin (Klinipath, Duiven, the Netherlands) was performed before dehydration and mounting using Permount (Thermo Fisher Scientific, Cheshire, UK).

All microscopic images were acquired using IP-Lab for Macintosh software (Scanalytics Inc., Fairfax, VA, USA) in combination with a monochrome CCD camera (Retiga SRV, 1392 × 1040 pixels) and a RGB filter (Slider Module; QImaging, Burnaby, BC, Canada) attached to a motorized microscope (Leica DM 6000, Wetzlar, Germany). For comparison between TRIB3 and pimonidazole expression, whole tumor sections were scanned with a 10× objective at 100× magnification [33]. The individual colors (DAB (brown) and hematoxylin (blue) signals) were extracted and unmixed from the bright fields images [33].

### Treatment of cells

For anoxia experiments, treatments included addition of 1.2 U/ml oxyrase (Oxyrase, Inc., Mansfield, OH, USA), a reagent that removes all oxygen from culture medium [34,35] or 100 μM CoCl_2 _(Sigma, Zwijndrecht, the Netherlands), a hypoxia mimetic, to the standard culture medium. The effect of CoCl_2 _on HIF-1α protein levels stabilization was confirmed by performing a standard western blot analysis [36] with minor adjustments using mouse anti-HIF-1α (BD Biosciences, Erembodegem, Belgium) antibody and the effect on HIF-1 activity by measuring CAIX expression on mRNA level.

For less than 0.01% O_2 _exposure, MCF7 cells were transferred to a hypoxic culture chamber (MACS VA500 microaerophilic workstation, Don Whitley Scientific, West Yorkshire, UK) as described previously [32].

For 0.1% to 0.5% O_2 _exposure MDA-MB-231 cells were transferred to a hypoxic culture chamber (H35 hypoxystation, Don Whitley Scientific, West Yorkshire, UK).

In nutrient starvation experiments, either glucose-free DMEM including the same additions as the standard culture medium, or the standard culture medium in which the NEAA were omitted, were used. To induce ER-stress, 0.5 μM thapsigargin (Sigma, Zwijndrecht, the Netherlands) was added to the standard culture medium.

### Cell proliferation assay

To measure cell survival we used the CellTiter 96^® ^AQ_ueous _One Solution Cell Proliferation Assay (MTS, Promega Benelux BV, Leiden, the Netherlands). Assays were performed in a 96-well plate format in which cells were transfected with siRNA 24 hours after seeding. Treatment started 24 hours after transfection. Directly after treatment for 48 hours 20 μl of MTS solution was added to 100 μl cell culture medium per well. After three hours of incubation with MTS absorbance at 492 nm was measured using a Multiskan Ascent Photometric plate reader (Labsytems, Helsinki, Finland).

### Statistical analyses

Statistical analyses were carried out using SPSS 10.0.5 software (SPSS Benelux BV, Gorinchem, the Netherlands). Normality of distribution of variables was tested using Kolmogorov-Smirnov testing. Differences in *TRIB3 *expression in cells with different treatments were evaluated using analysis of variance (ANOVA) and *post-hoc *Tukey's testing. Differences in levels of *TRIB3 *expression in samples from patients categorized by clinicopathological characteristics, used as grouping variables, were assessed with non-parametric Mann-Whitney U tests (for two groups) or with Kruskall-Wallis tests (for more than two groups) where appropriate. Non-parametric correlations were established using Spearman Rank correlation testing. Disease free survival (DFS) time (defined as the time from surgery until diagnosis of recurrent or metastatic disease) and overall survival (OS) time (defined as the time between date of surgery and death by any cause) were used as follow-up endpoints. Survival curves were generated using the method of Kaplan and Meier, after patients were categorized by *TRIB3 *expression in either two or three equally sized groups, thus either at the p50, or at the p33 and p66. Equality of survival distributions was tested using log-rank testing, with Mantel-Cox test for trend when more than two groups were analyzed, and using Cox univariate and multivariable regression analyses. Variables were selected for the multivariable survival analyses by backward stepwise selection, with removal testing based on the probability of the likelihood-ratio statistic, at a *P *> 0.10. Two-sided *P*-values below 0.05 were considered to be statistically significant. Cases with more than 96 months of follow up were censored at 96 months, because of the rapidly declining number of patients thereafter, although data on some patients was available for up to 169 months after primary surgery. This censoring was done because after a certain period of observation patients are frequently redirected to their general practitioner for checkups and mammography and cease to be among the outpatients of the breast cancer clinics. Further inclusion of the small remaining groups in statistical analyses would be non-informative. Additionally, the data met the proportional hazard assumption, and hazard ratios did not change over time.

## Results

### *TRIB3 *mRNA association with a poor prognosis in human breast cancer

To investigate whether the variation in *TRIB3 *expression is relevant for prognosis in breast cancer, *TRIB3 *mRNA levels were measured in 247 tumor samples from breast cancer patients. The *TRIB3 *levels were log-normally distributed among the patient samples, with 26 of 247 (10.5%) samples in which *TRIB3 *levels were not detectable. *TRIB3 *levels showed no association with most clinicopathological characteristics (Table 1). *TRIB3 *mRNA levels were only significantly different in tumors that were not of ductal or lobular histology.

**Table 1:** Associations of *TRIB3 *expression levels with clinicopathological factors.

Variable	***n *= 247**^**a**^	Median (*10^-3 ^TRIB3/HPRT)	Interquartile range (*10^-3 ^TRIB3/HPRT)	*P*
Age (years)				
< 50	58	8.6	36	
≥50	189	6.6	26	0.456^b^
Menopausal status				
Premenopausal	65	9.4	34	
Postmenopausal	182	6.6	27	0.500^b^
Nodal category				
Negative	100	9.0	31	
< 4 nodes	79	4.7	18	
≥4 nodes	39	16	46	0.057^c^
Tumor type				
Ductal	146	8.6	35	
Lobular	28	13	25	
Other (mixed/unknown)	73	2.7	18	0.007^c^
Tumor size				
pT1	61	5.2	28	
pT2	135	7.1	34	
pT3/4	47	6.9	22	0.616^c^
Histological grade				
I	11	5.1	17	
II	70	6.4	36	
III	88	8.5	31	0.729^c^
Estrogen receptor (fmol/mg protein)				
< 10	84	7.2	30	
≥10	159	5.8	27	0.895^b^
Progestrone receptor (fmol/mg protein)				
< 10	103	7.1	32	
≥10	141	6.6	23	0.663^b^

^a^Due to missing values, numbers do not always add up to 247.

^b ^*P *for Mann-Whitney U test.

^c ^*P *for Kruskal-Wallis test.

**Figure 1:**
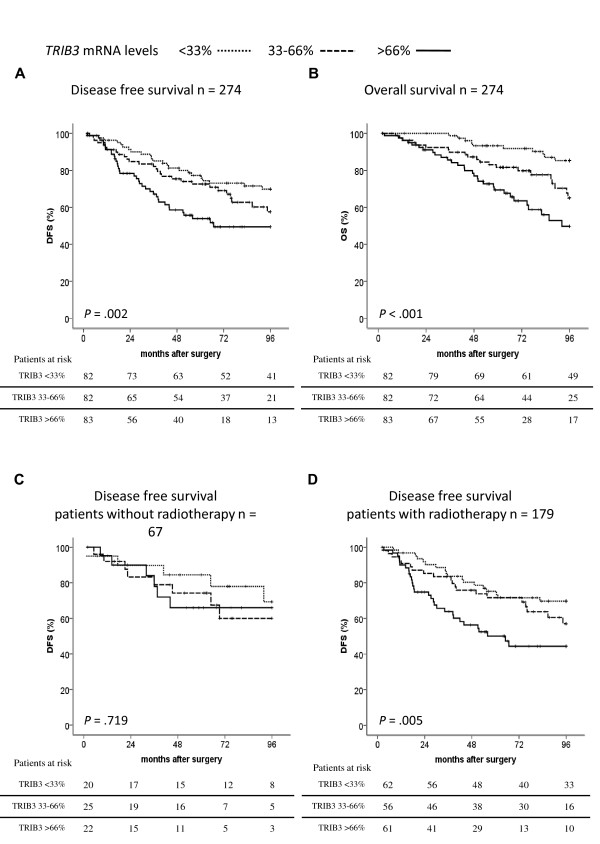
**TRIB3 expression is associated with poor prognosis in breast cancer**. Patients where divided into three groups based on their *TRIB3 *mRNA levels; less than 33% (dotted line), 33% to 66% (dashed line) and more than 66% (solid line). Kaplan-Meier plots of **(a) **disease-free survival (DFS; *P *= 0.002) and **(b) **overall survival (OS; *P *< 0.001) in breast cancer patients (*n *= 274) and DFS **(c) **in patients with mastectomy without further radiotherapy (*n *= 67, *P *= 0.719) and **(d) **in patients that received postoperative radiotherapy (*n *= 179, *P *= 0.005).

HPRT, hypoxanthine-guanine phosphoribosyltransferase;

When the patients were divided into three equally sized groups based on the level of *TRIB3 *mRNA in the primary tumor the group with the lowest amount of *TRIB3 *expression had a mean DFS of 80 months (95% confidence interval (CI) = 74 to 86 months), whereas the group with an average amount of *TRIB3 *had a mean DFS of 74 months (95% CI = 67 to 81 months) and the group with high levels of *TRIB3 *had a mean DFS of 63 months (95% CI = 55 to 71 months; *P *= 0.002, Figure 1a). Patients with the lowest amount of *TRIB3 *expression had a mean OS of 91 months (95% CI = 88 to 94 months), whereas those with an intermediate amount of *TRIB3 *had a mean OS of 83 months (95% CI = 77 to 89 months) and those with high levels of *TRIB3 *had a mean OS of 74 months (95% CI = 67 to 80 months; *P *< 0.001, Figure 1b). Similar differences were found after division of the patients in two equal groups (DFS: hazard ratio (HR) = 2.15, 95% CI = 1.39 to 3.31, *P *< 0.001; OS: HR = 3.48, 95% CI = 2.00 to 6.06, *P *< 0.001; Table 2), and when *TRIB3 *was entered as a continuous factor after log-normalization in a univariate Cox regression analysis (not shown). In multivariate Cox regression analyses, *TRIB3 *remained statistically significantly associated with both DFS and OS after correction for other clinicopathological parameters (Table 2).

**Table 2:** Cox uni- and multivariate analyses of disease-free survival (DFS) and overall survival (OS) in all patients (*n *= 247).

	DFS				OS			
Factor	Univariate analysis		Multivariate analysis		Univariate analysis		Multivariate analysis	
	*P*-value	**HR (95% CI)**^**a**^	*P*-value	**HR (95% CI)**^**a**^	*P*-value	**HR (95% CI)**^**a**^	*P*-value	**HR (95% CI)**^**a**^
Age (years)	0.036		0.008		0.939			
41-55 *vs*. ≤40		0.59 (0.30-1.17)		0.38 (0.18-0.79)		1.10 (0.44-2.76)		
56-70 *vs*. ≤40		0.58 (0.31-1.12)		0.53 (0.27-1.06)		0.93 (0.38-2.28)		
> 70 *vs*. ≤40		0.30 (0.13-0.69)		0.20 (0.07-0.55)		0.89 (0.33-2.41)		
Menopausal status	0.024				0.255			
Post- *vs*. premenopausal		0.77 (0.61-0.96)				0.73 (0.42-1.25)		
Tumor size	0.006		0.023		0.111			
pT2 *vs*. pT1		2.15 (1.17-3.95)		2.36 (1.20-4.65)		1.59 (0.82-3.07)		
pT3+4 *vs*. pT1		2.81 (1.40-5.62)		2.87 (1.30-6.37)		2.23 (1.04-4.77)		
Histological grade	0.091				0.768			
II *vs*. I		1.54 (0.36-6.58)				1.38 (0.32-5.94)		
III *vs*. I		2.80 (0.68-11.6)				1.76 (0.42-7.44)		
Number of involved lymph nodes	0.001		0.013		0.005		0.006	
1-3 *vs*. node-negative		1.51 (0.89-2.56)		1.41 (0.81-2.44)		1.39 (0.73-2.65)		1.53 (0.80-2.93)
≥4 *vs*. node-negative		3.29 (1.84-5.88)		2.63 (1.38-5.00)		3.32 (1.67-6.64)		3.17 (1.56-6.43)
Estrogen receptor status^b^	0.245				0.845			
Positive *vs*. negative		0.77 (0.50-1.19)				0.95 (0.55-1.63)		
Progestrone receptor status^b^	0.431				0.650			
Positive *vs*. negative		0.84 (0.55-1.29)				0.89 (0.53-1.48)		
TRIB3	< 0.001		0.009		< 0.001		< 0.001	
Higher vs. lower than median		2.15 (1.39-3.31)		1.90 (1.17-3.08)		3.48 (2.00-6.06)		3.90 (2.06-7.37)
								

^a ^Hazard ratio (95% confidence interval (CI))

^b ^positive defined as > 10 fmol/mg protein

In exploratory subgroup analyses, we found that the prognostic value of *TRIB3 *mRNA expression was limited to those patients receiving radiotherapy as part of their primary treatment. In those patients that had a mastectomy without further radiotherapy, no relation between *TRIB3 *and DFS was seen (*P *= 0.719, *n *= 67, Figure 1c). Postoperative radiotherapy was given to 179 patients (to the breast after an incomplete resection or after breast-conserving treatment, or parasternal radiotherapy when the tumor was medially localized). In this group, *TRIB3 *was highly significantly associated with DFS (*P *= 0.005, *n *= 179, Figure 1d). We did not find a correlation between *TRIB3 *mRNA expression and local/regional or distant control in the radiotherapy treated group (*P *= 0.13, *P *= 0.11 and *P *= 0.17 respectively), neither in the group not treated with radiotherapy (*P *= 0.91 *P *= 0.75 and *P *= 0.84, respectively).

### *TRIB3 *mRNA is induced by a variety of stresses

**Figure 2:**
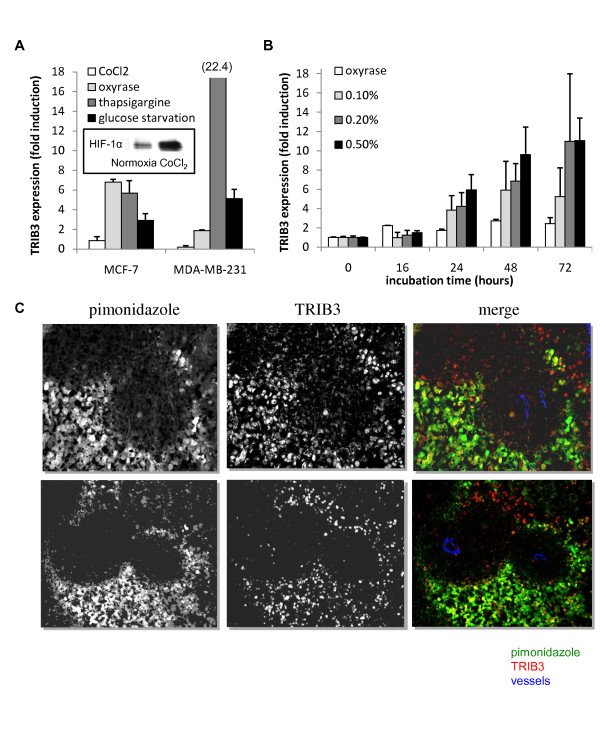
**TRIB3 is induced by cell stress and is expressed in hypoxic areas**. **(a) **Fold induction of *TRIB3 *mRNA expression (mean + standard deviation (SD)) after 24 hours incubation with hypoxia mimetic (CoCl_2_, inhibitor of the degradation of HIF-1, see insert), anoxia (oxyrase), estrogen receptor (ER) stress (thapsigargin) or nutrient starvation in the ER positive MCF7 and the ER negative MDA-MB-231 cell lines. Each measurement was performed at least in triplicate. **(b) **Fold induction of *TRIB3 *mRNA expression (mean + SD) after incubation of MDA-MB-231 cells at increasing oxygen concentrations (oxyrase (~0%), 0.1%, 0.2% and 0.5% oxygen) compared with normoxia. Each measurement was performed at least in triplicate. **(c) **Triple fluorescent staining in two different xenografts of MDA-MB-231 cells for TRIB3 (red), pimonidazole (green), and 9F1 (blue) showing colocalization of TRB3 and hypoxia, distal from blood vessels.

To test the effect of different stresses on TRIB3 mRNA expression we used the estrogen receptor positive MCF7 and estrogen receptor negative MDA-MB-231 breast cancer cells. Anoxia exposure was performed using oxyrase, HIF-1 protein was stabilized using CoCl_2_, ER stress was induced by treating the cells with 0.5 μM thapsigargin (an inhibitor of endoplasmic reticular Ca^2+^-ATPase), and nutrient starvation was achieved by deprivation of the medium from either glucose or NEAA. After different incubation periods *TRIB3 *mRNA levels were measured by RT-qPCR. Figure 2a shows the results of 24 hours of incubation. *TRIB3 *expression was increased in breast cancer cells after 24 hours of anoxia (*P *< 0.05, Figure 2a). However, incubation with CoCl_2_, which indeed stabilized HIF1α protein confirmed by western blot (see insert in Figure 2a) and induced HIF1-dependent transcription measured by CAIX mRNA expression (fold induction ranging from 1.3 to 4.3 for MDA-MB-231 and 35.7 to 65.4 for MCF7 cells) did not induce *TRIB3 *mRNA. Thapsigargin induced a significant increase in *TRIB3 *mRNA levels within two hours, which was further extended after up to 24 hours of incubation (*P *< 0.01, Figure 2a). After MCF7 cells were starved for glucose for 24 hours, *TRIB3 *expression was significantly increased (*P *< 0.01, Figure 2a). After omission of NEAA from the growth medium, a statistically significant increase in *TRIB3 *mRNA was detected at 48 hours (*P *< 0.01, data not shown).

### TRIB3 induction by hypoxia in breast cancer cells, xenografts and breast cancer tissues

To specify the oxygen dependence of *TRIB3 *up-regulation MDA-MB-231 cells were cultured under controlled oxygen concentrations of 0.1, 0.2, and 0.5%. A time dependent up-regulation of *TRIB3 *mRNA compared with *HPRT *was seen at all tested oxygen tensions after 24 hours. More severe oxygen deprivation does not lead to a higher induction of *TRIB3 *compared with intermediate hypoxia (Figure 2b).

Next, to assess whether the oxygen dependence of TRIB3 expression could also be observed in tissues, frozen sections of MDA-MB-231 human breast cancer cell xenografts grown on nude mice were triple stained with fluorescent anti-pimonidazole, anti-TRIB3, and anti-endothelial antibodies. TRIB3 staining was specifically seen in the hypoxic regions of the tumor, distant from the blood vessels (Figure 2c).

**Figure 3:**
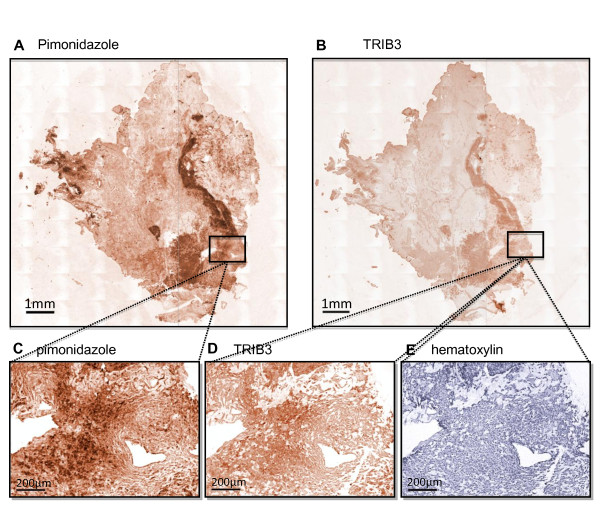
**TRIB3 colocalizes with pimonidazole in human breast cancer tissue**. Results after linear unmixing of scanned images from immunohistochemical stainings of **(a) **TRIB3 (DAB, brown) and **(b) **pimonidazole (DAB, brown), and single images from immunohistochemical stainings at 100× magnification of **(c) **TRIB3 (DAB, brown), **(d) **pimonidazole (DAB, brown) and **(e) **hematoxylin (blue), in human breast cancer tissue. See methods for details of unmixing and scanning.

Next, we assessed TRIB3 staining in breast cancer tissue from three patients that had received pimonidazole prior to biopsy. In all three tumor tissues, large pimonidazole positive hypoxic areas were found, often around necrotic cores. TRIB3 was almost exclusively expressed in these hypoxic areas (Figure 3). Excellent co-localization was seen between pimonidazole and TRIB3 within the same regions at higher magnification (Figures 3c to 3e). Thus, in human breast cancer tissues, TRIB3 is up-regulated in hypoxic regions of the tumor, similar to that seen in xenograft samples shown above.

### *TRIB3 *is induced through the unfolded protein response via PERK, ATF4 and CHOP

**Figure 4:**
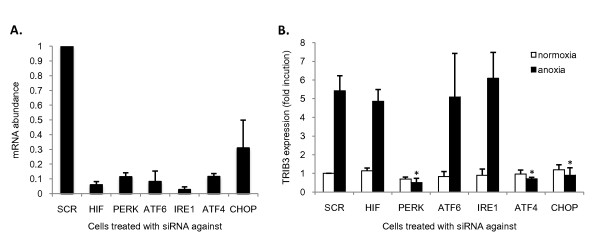
**TRIB3 is induced through the PERK/ATF4/CHOP pathway of the unfolded protein response**. **(a) **mRNA abundance of the specific genes targeted by the specific siRNAs (mean + standard deviation (SD; *n *= 3). **(b) **Fold induction of *TRIB3 *expression (mean + SD) after anoxia (< 0.01% O_2_) in MCF7 cells with knockdown of scrambled control (SCR), *HIF1*, *PERK*, *ATF6*, *IRE1*, *ATF4 *or *CHOP *gene by siRNAs. *Significantly different from control (SCR) under anoxia, *P *< 0.01, each measurement was performed in triplicate.

Tumor cells respond to oxygen deprivation by, among others, stabilization of HIF-1 and activation of the UPR, which consists of three parallel pathways (PERK, IRE1, and ATF6) [8,37]. To assess through which of these pathways *TRIB3 *is up-regulated after anoxia, *TRIB3 *mRNA levels were measured in MCF7 cells in which the different pathways were silenced by gene knockdown [32]. For all genes used an efficient knockdown for that specific gene was seen (Figure 4a). In the control cells, *TRIB3 *mRNA was strongly induced after culturing under anoxic conditions (< 0.01% O_2_; Figure 4b). Knockdown of HIF-1α did not alter the anoxia induction of *TRIB3 *expression at any time point, which is in line with our results in breast cancer cells after CoCl_2 _incubation. ATF6 or IRE1 knockdown also did not have an effect on the anoxia-induced up-regulation of *TRIB3*. In contrast, knockdown of PERK, ATF4 or CHOP completely inhibited the up-regulation of *TRIB3 *(*P *< 0.01).

### TRIB3 mRNA expression is not a hypoxia marker

Expression levels of other hypoxia and UPR-associated genes were also determined in the breast cancer patient cohort. No correlation between *TRIB3 *mRNA expression and the hypoxia markers VEGF, PAI-1, CAIX, or MIF was observed (Spearman correlation coefficient (R_s_) = 0.19, *P *= 0.05, R_s _= -0.38, *P = *0.59, R_s _= 0.78, *P = *0.25 and R_s _= 0.11, *P = *0.08, respectively). The expression of ATF4, the main regulator of TRIB3, was borderline significantly correlated with *TRIB3 *mRNA expression (*P *= 0.05) with a low correlation coefficient of 0.18. Another UPR-associated gene GRP78/BiP was not correlated with *TRIB3 *expression (R_s _= 0.12, *P *= 0.15).

### Knockdown of *TRIB3 *results in a increased hypoxia sensitivity

**Figure 5:**
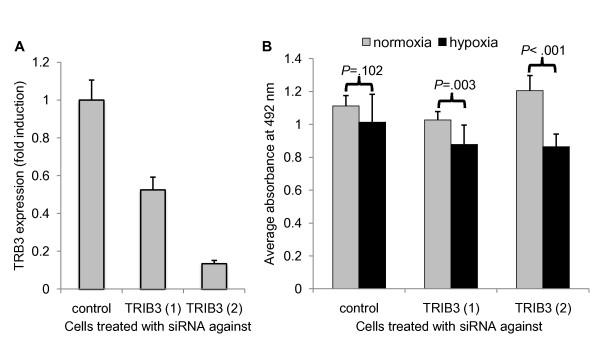
**Knockdown of TRIB3 results in lower cell survival under hypoxic conditions**. **(a) **Fold induction of *TRIB3 *expression levels (mean + standard deviation (SD)) in MDA-MB-231 cells after transfection with negative control siRNA and two siRNAs against *TRIB3 *transcript. Each measurement was performed in triplicate. **(b) **Average absorbance at 492 nm (+ SD) measured by MTS assay of cells cultured under normal culture conditions and 0.1% oxygen. Each condition was measured in 10 independent wells.

MDA-MB-231 cells were used for siRNA-mediated knockdown of *TRIB3*. Two different siRNAs against *TRIB3 *were used and *TRIB3 *expression was compared with cells transfected with negative control siRNA. TRIB3 (1) siRNA led to a reduction in *TRIB3 *expression of 48%, and TRIB3 (2) siRNA led to a reduction of 87% (Figure 5a). Next, we sought to establish whether TRIB3 is associated with poor prognosis solely as a marker for hypoxia, or that it has a functional role in hypoxia resistance of tumor cells. To this end, MDA-MB-231 cells were transfected with the above mentioned TRIB3 siRNAs and cultured under normoxic and hypoxic conditions. In cells transfected with control siRNA, the total activity of cells measured by MTS assay cultured for 48 hours at 0.1% did not differ significantly from cells cultured in standard conditions (Figure 5b). In cells transfected with the two different siRNAs against *TRIB3 *the MTS assay readout was significantly (*P *= 0.003 and *P *< 0.001 for siRNA TRIB3 (1) and (2), respectively) diminished after hypoxia. Noteworthy, the siRNA that has the greatest knockdown effect on *TRIB3 *mRNA levels also had the greatest effect on total cell activity after hypoxia.

## Discussion

Here, we find that TRIB3 is associated with poor prognosis of breast cancer patients, independent of other clinicopathological characteristics. TRIB3 is expressed in hypoxic areas of both breast cancer xenografts and human breast tumor tissues. We found that this *TRIB3 *up-regulation is induced by ER stress, hypoxia and nutrient starvation, and is dependent on the PERK pathway of the UPR. *TRIB3 *induction is most pronounced at a moderate oxygen concentration and TRIB3 seems involved in hypoxia response of tumor cells.

Tribbles was originally identified as a delayer of mitosis in *Drosophila Melanogaster *[38-40]. One of the three human homologs, TRIB3, has recently been described to be involved in regulation of the cell cycle regulator cdc25A in human cells [41]. Furthermore, TRIB3 has a role in insulin sensitivity and diabetes [10,42-44] and has also been described to interact as a scaffold protein in multiple signaling cascades, including v-akt murine thymoma viral oncogene homolog 1 (PKB/Akt) [10,13] but also MAPK [11] pathways. This apparent involvement of TRIB3 in tumor cell proliferation and/or survival and growth factor receptor signaling cascades combined with its role in hypoxia and cell stress pathways [9,13-15,45,46] originally spurred us to investigate its role in breast cancer progression. Our results presented here show that TRIB3 could be involved in the hypoxia response of breast cancer cells; it is induced by cell stressors including hypoxia, ER stress, and nutrient starvation in breast cancer cells, which is in line with earlier observations in other cell types [9,13-15,45,46]. We confirmed here that TRIB3 is part of the UPR [9,12], more specifically the PERK/ATF4/CHOP pathway. We find that knockdown of *TRIB3 *results in cells that are more sensitive to hypoxia. One pathway involved in hypoxia tolerance of tumor cells is the UPR [32]. Tumor cells utilize this pathway to counter cell stresses like hypoxia, most likely through induction of autophagy [32]. By "self-eating" of cellular components, cells can provide for their own energy and constituents, thereby surviving prolonged periods of severe hypoxia [32]. TRIB3 is known to inhibit PKB/Akt [10], a putative link between the UPR and autophagy [16].

Importantly, hypoxic cells are particularly refractory to treatment and tend to have an increased metastatic potential [1,3]. Further, the hypoxia and nutrient starvation-induced UPR-pathway is important for cancer treatment efficacy [21,32,47,48] and therefore an interesting new target for therapy. In comparison with HIF-1, which is activated over a wide range of oxygen concentrations of around 2%, maximum activation of the UPR requires exposure to more severe hypoxia (reviewed in [49]). Nevertheless, activation of the UPR has been shown to protect tumor cells against hypoxia-induced cell death both *in vitro *and *in vivo *over a range of oxygen concentrations [47,48,50]. We are the first to describe the specific oxygen tensions that induce *TRIB3*, and found that up-regulation of *TRIB3 *does not increase with decreasing oxygen tensions. These results indicate that the up-regulation of *TRIB3 *under moderate hypoxia is at least partly due to other pathways than the UPR. PI3K is already described to up-regulate *TRIB3 *expression [13] and could be responsible for the up-regulation of *TRIB3 *at the more physiological moderate hypoxia, rather than at severe hypoxia (i.e. anoxia). Severe hypoxia is mainly found in perinecrotic areas within solid tumors and probably has less influence on prognosis and treatment sensitivity than areas with more moderate hypoxia. Within the moderate hypoxic tumor regions cells can adapt, and are eventually more likely to reoxygenate and/or metastasize as they are more proximal to blood vessels.

Here, we show that TRIB3 expression can independently predict disease outcome in human breast cancer patients. The importance of TRIB3 in cancer is supported by the finding that TRIB3 is also involved in the prognosis of colorectal cancer patients [17]. The data suggest that TRIB3 is induced by hypoxia in an ATF4 dependent manner and supports hypoxia sensitivity, but from the measurements of other hypoxia markers we find that *TRIB3 *mRNA abundance from tumor biopsies is not merely a reflection of tumor hypoxia. This suggests that *TRIB3 *marks or supports tumor aggressiveness rather than reflecting hypoxia itself. Furthermore, the predictive value of *TRIB3 *expression in our study was specifically significant in the patient group that received radiotherapy. Combined with the observation that TRIB3 is mostly expressed in the therapy resistant hypoxic areas of breast tumors this indicates that the prognostic role of TRIB3 could indeed be a consequence of therapy resistance. When this would be solely due to radiotherapy resistance one would expect also a correlation with loco-regional control but this was not the case. However, a correlation with distant control could also not be found, probably both due to low number of events and small group sizes. In addition, as this was a non-randomized retrospective analysis, any predictive value of *TRIB3 *mRNA remains to be confirmed in a larger prospective trial.

## Conclusions

Examining *TRIB3 *expression in human breast tumor material revealed that there was an independent association with poor prognosis of breast cancer patients. A relation of TRIB3 with hypoxia was seen by the co-localization with the hypoxia marker pimonidazole in both breast cancer xenografts and human breast tumor tissue. We found that *TRIB3 *up-regulation after hypoxia, ER stress and nutrient starvation also holds true for breast cancer cells and is HIF-1 independent and UPR dependent. *TRIB3 *up-regulation after hypoxia was found to be most pronounced at physiological intermediate hypoxia, in contrast to most UPR-induced proteins. Finally, knockdown of *TRIB3 *revealed an effect on hypoxia response of breast cancer cells. In combination these results indicate that TRIB3 might be associated with tumor cell survival under prolonged intermediate hypoxic stress. This hypothesis warrants further experiments in other (breast cancer) cell lines applying additional knockdown, possibly with rescue, and/or overexpression techniques. Furthermore, the involvement of TRIB3 in multiple important signaling pathways makes it an interesting target for cancer therapy. Further research will provide more insight into the mechanisms of action and possibilities of intervention.

## Abbreviations

ANOVA: analysis of variance; ATF4: activating transcription factor 4; CHOP: C/EBP homologous protein; CI: confidence interval; DFS: disease free survival; DMEM: Dulbecco's Modified Eagle Medium; ER: endoplasmic reticulum; HIF-1: hypoxia inducible factor 1; HPRT: hypoxanthine-guanine phosphoribosyltransferase; HR: hazard ratio; IRE1: inositol-requiring 1; MAPK: mitogen activated protein kinase; NEAA: nonessential amino acids; NF: nuclear factor; OS: overall survival; PERK: PRKR-like endoplasmic reticulum kinase; PBS: phosphate buffered saline pH 7.4; PKB/Akt: v-akt murine thymoma viral oncogene homolog 1; RT-qPCR: reverse transcriptase: quantitative polymerase chain reaction; siRNA: small interfering RNA; TRIB3: Tribbles homolog 3; UPR: unfolded protein response.

## Competing interests

The authors declare that they have no competing interests.

## Authors' contributions

MW performed laboratory experiments, data analysis and participated in the experimental design and drafting of the manuscript. JB participated in the design and coordination of the research and assisted in xenograft assays. BS contributed to the interpretation of the data and critical revised the manuscript. IDN participated in the immunohistochemical stainings and the interpretation of these data. HWML participated in the collection of the patient material and data. JAR and MAV participated in the collection of patient material containing pimonidazole. JJTMH participated in RT-qPCR experiments and interpretation of the results. KMR carried out the MCF-7 knockdown experiments and critically revised the manuscript. FCGJS participated in the design and coordination of the research and assisted in the patient material collection. PNS participated in the design and coordination of the research, performed the statistical analysis and helped to draft the manuscript. All authors read and approved the final manuscript.
